# Antiphospholipid syndrome with mesenteric vein thrombosis and hepatic nodular regenerative hyperplasia in a child: A case report

**DOI:** 10.1097/MD.0000000000028105

**Published:** 2021-12-03

**Authors:** Seung Hyung Han, Kyung Duk Park, Soon Chul Kim

**Affiliations:** Department of Pediatrics, Jeonbuk National University Medical School and Hospital, Research Institute of Clinical Medicine of Jeonbuk National University - Biomedical research Institute of Jeonbuk National University Hospital, Jeonju, Korea.

**Keywords:** antiphospholipid syndrome, nodular regenerative hyperplasia, thrombosis

## Abstract

**Rationale:**

Hepatic nodular regenerative hyperplasia (NRH), a nonspecific change in the liver parenchyma, is very rare in children. Hepatic microvascular changes may be the cause, as these vascular changes are uncommon in children. Antiphospholipid syndrome (APS), an autoimmune disease characterized by vascular thromboembolism, is extremely unusual in children.

**Patient concerns:**

A 13-year-old girl who presented with abdominal pain and elevated liver enzymes was transferred to our hospital. Abdominal computed tomography and magnetic resonance imaging showed a massive mesenteric venous thrombus and a malignant mass with liver metastasis.

**Diagnoses:**

Her immunological profile was positive for antinuclear antibodies (ANA) at a titer of 1/160 (nucleolar pattern), anticardiolipin antibodies (aCL) immunoglobulin G, and anti-histone antibody. A liver biopsy revealed hepatic NRH.

**Interventions:**

The patient was initially started on heparin upon hospitalization and switched to warfarin and a vitamin K antagonist and continued treatment with international normalized ratio monitoring.

**Outcomes:**

Her symptoms improved after 9 months of anticoagulation therapy.

**Lessons:**

In the presence of hepatic NRH or vascular thrombosis in children, we recommend that APS be differentially diagnosed using lupus anticoagulant and aCL and appropriate management be implemented.

## 1 Introduction

Hepatic nodular regenerative hyperplasia (NRH) is extremely uncommon in children and may be caused by hepatic vascular disorders associated with various systemic diseases. The etiology is described as a hepatocytic hyperplastic reaction in which abnormalities occur in the normal flow of the hepatic microvasculature.^[1]^ Depending on the degree of involvement, NRH can be asymptomatic or exhibit very diverse symptoms from mild liver enzyme elevations to portal hypertension.

Antiphospholipid syndrome (APS) is rare in children and characterized by systemic thrombosis with positive antiphospholipid antibodies (aPL), such as lupus anticoagulant (LA) or anticardiolipin antibodies (aCL).^[2]^ APS can occur secondary to other diseases, such as systemic lupus erythematosus (SLE); however, this case is a primary APS occurring independently. The hypercoagulability state caused by APS can affect various organs in the abdominal cavity, such as the liver, intestine, spleen, and pancreas.^[3]^ We found hepatic nodules and a massive mesenteric vein thrombosis on an abdominal computed tomography (CT) scan of a 13-year-old girl who presented with abdominal pain and elevated liver enzymes. On histological examination and further evaluation, NRH, multiple venous thromboses, and APS were diagnosed. All 3 diseases improved through the treatment of APS. This patient was diagnosed in May 2020 and had not been previously diagnosed. We report this case along with a literature review.

## 2 Case report

A 13-year-old girl presented with lower abdominal pain since the preceding week. On admission, her height was 165.6 cm (90th–95th percentile), weight was 61 kg (75th–90th percentile), vital signs were stable, and body temperature was 36.7°C. Physical examination revealed a soft and flat abdomen, normoactive bowel sounds, and tenderness of the lower left abdomen. The liver was palpable to two fingers below the subcostal margin and without splenomegaly.

**Figure 1:**
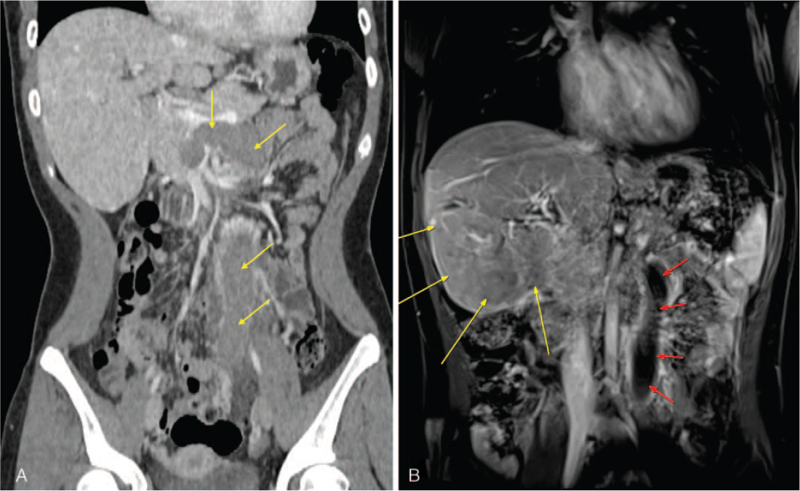
(A) Initial computed tomography with contrast showing inferior mesenteric vein (IMV) thrombosis in the portal phase (4 thin arrows). (B) Initial abdominal magnetic resonance image displaying nodular regenerative hyperplasia lesion (long thick arrows) and IMV thrombus (short, thin arrows).

The patient underwent abdominopelvic CT and magnetic resonance imaging (MRI) at our hospital due to abdominal pain. CT revealed a massive thrombus from the superior rectal vein to the inferior mesenteric vein (IMV) confluence, the collateral vessel developed in the pelvic cavity, and a focal thrombus was seen in some IMV branches without visible thrombosis in the portal, splenic, renal, and ovarian veins (Fig. 1A). On abdominal MRI, liver segments 5 and 6 appeared to have atrophic changes, while a large nodular lesion was suspected in the left lateral lobe and segment 4, a finding of NRH (Fig. 1B). Further evaluation was performed using positron emission tomography–CT as nodules and masses in the liver were suspected, especially in segments 4 to 6, that showed a similar degree of metabolism to the liver parenchyma.

**Figure 2:**
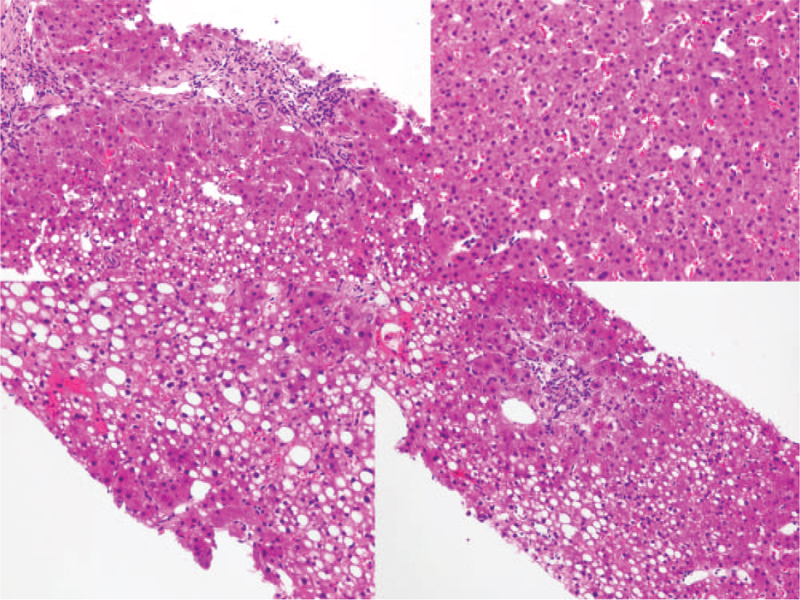
A slide at 200 × magnification showing a reduced number of interlobular bile ducts with ductular proliferation, centrilobular cholestasis, macrovesicular fatty change, mild proto-periportal inflammation, mild lobular inflammation, and sinusoidal dilatation.

At the time of admission, the laboratory results showed hemoglobin, 12.3 g/dL; white blood cell count, 8970/μL; platelet count, 229,000/μL; erythrocyte sedimentation rate, 45 mm/h; C-reactive protein, 12.74 mg/L; alanine aminotransferase, 57 IU/L; aspartate aminotransferase, 63 IU/L; gamma-GT, 227 IU/L; and alkaline phosphatase, 307 IU/L. In coagulation tests, the prothrombin time (PT) and activated partial thromboplastin time (APTT) were normal, but fibrinogen degradation products (53.76 μg/mL) and D-dimer (21.742 mg/L) levels were elevated. Protein C Ag, protein C activity, and protein S Ag levels were normal, but protein S activity was decreased (36%, normal: 55–123%). Serological tests for cytomegalovirus and hepatitis A, B, and C were negative. The initial immunological profile was positive for antinuclear antibodies (ANA) at a titer of 1/160 (nucleolar pattern). It was also positive for aCL immunoglobulin G (IgG) but negative for aCL immunoglobulin M (IgM) and anti-beta2-glycoprotein. No LA, aPL IgG, or aPL IgM was detected. All laboratory tests were repeated after 3 months, at which time the aCL IgG was still positive. Anti-histone antibodies were positive in the repeated tests. Thus, APS was diagnosed. Liver biopsy was performed, and the findings showed a reduced number of interlobular bile ducts with ductular proliferation, centrilobular cholestasis, macrovesicular fatty changes, mild proto-periportal inflammation, mild lobular inflammation, sinusoidal dilatation, and cytokeratin (CK)7 and CK19 positivity in bile duct cells. These results were consistent with hepatic NHR (Fig. 2).

Initially, we administered intravenous heparin while monitoring the APTT levels. After 5 days, the patient was switched to oral warfarin and PT monitoring was started. We started intravenous heparin with a bolus dose of 80 units/kg infused over 10 minutes then continued with a maintenance rate of 20 units/kg/h. Per guidelines, we adjusted the heparin dose according to the patient's APTT. The target APTT range was 60 to 85 seconds. On oral warfarin, a loading dose of 10 mg (approximately 0.2 mg/kg) was given, and PT monitoring was started. The warfarin dose was adjusted by outpatient INR levels (target level, 2.0–3.0). Upon discharge, the dose was further adjusted to 2.5 mg/day. After discharge, follow-up INR was performed every 5 days, and the warfarin dose was increased 3 times, each by 50% until target INR was reached at the maintenance dose of 6 mg/day (0.1 mg/kg). Our patient was maintained on the same dose and INR was sustained at 2.0 to 3.0 for more than 3 months as an outpatient.

**Figure 3:**
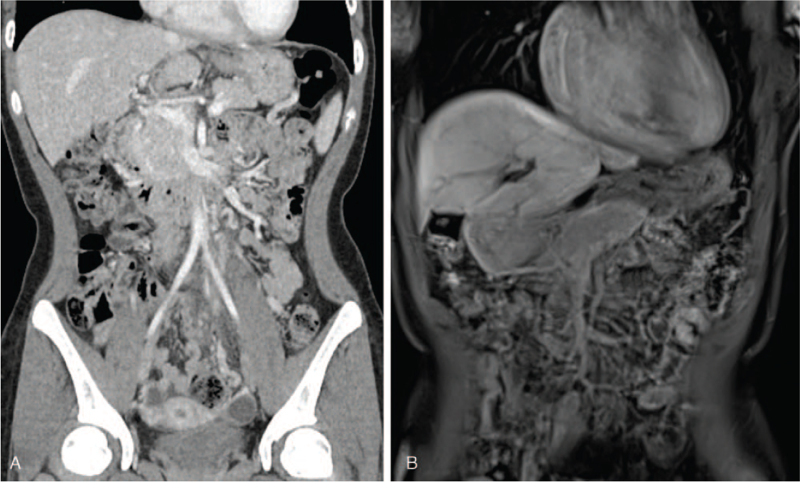
Computed tomography (CT) and magnetic resonance imaging after 9 months of warfarin treatment. (A) Diffuse thrombosis in inferior mesenteric vein (IMV) confluence is almost resolved on CT. (B) IMV thrombosis is improved. There is a contour change in the liver, and the hyperplastic lesion is slightly decreased.

Abdominal CT and liver MRI were performed every 3 months, while the patient was on warfarin. On follow-up, abdominal CT and liver MRI revealed alleviated hyperplasia of liver segment 5, and while the diffuse thrombosis in the IMV developed to a chronic thrombus, it had mostly resolved in the proximal portion, as opposed in the distal portion (Fig. 3). About a year after treatment, the patient tested negative for aCL. However, the patient experienced 2 side effects during treatment, dyspnea during hiking and pulmonary hypertension on echocardiography. Chest CT was performed, considering the possibility of thromboembolism-induced pulmonary hypertension, but pulmonary thromboembolism was not detected. Subsequently, echocardiography was done every 3 months, and although there was no improvement, no worsening of clinical symptoms occurred. Thus, the patient was continued on warfarin with outpatient follow-ups.

## 3 Discussion

NRH is characterized by a diffuse and non-fibrotic transformation of the hepatic parenchyma with vascular abnormalities. Hypercoagulability and thrombotic changes in small hepatic vessels caused by these vasculitis-causing diseases appear to play an important role.^[4,5]^ In this case, APS seemed to play a role, but neither portal vein thrombosis nor hepatic vein thrombosis was detected. Clinically, NHR can be asymptomatic or exhibit symptoms and complications associated with non-cirrhotic portal hypertension, such as thrombocytopenia, esophageal varices, ascites, and splenomegaly.^[6]^ Prognosis is commonly relative to the severity of the underlying disease, although a recent report correlates it to malignancies such as hepatocellular carcinoma.^[7]^

The diagnosis of NHR can be made using liver biopsy. Histological findings of nodules show hyperplastic hepatocytes in the center and atrophic cells at the edges without fibrosis. Furthermore, it has recently been reported that changes in CK7 and CK34 expression in hepatocytes and sinusoidal endothelial cells can cause portal hypertension, which is an important finding to aid in diagnosis.^[8,9]^ Imaging modalities can help diagnose the disease. Sonographic findings of NRH include small isoechoic nodules scattered in any lobe. Generally, CT images show low attenuated multiple nodules, although MRI is more useful than ultrasound or CT in detecting nodules. On T1-weighted images, NRH appears hyperintense while conversely isointense or hypointense on T2-weighted images.^[10]^ Our patient's MRI revealed that liver segments 5 and 6 appeared to have atrophic changes, while the left lateral lobe and segment 4 were suspected to have a large nodular lesion.

Venous thromboembolism (VTE) is rarely diagnosed in children, but reports are indicating it has been increasing recently.^[11]^ This is also related to an increase in risk factors for VTE, which range from genetic problems to acquired factors such as the presence of disease, immobility, and the use of a central catheter. The pathogenesis of thrombus development is described as the Virchow triad (venous stasis, endothelial damage, and hypercoagulable state); if one or more of the 3 conditions are present, the risk of VTE increases.^[12–14]^ In our patient, IMV thrombosis was confirmed by abdominal CT. The positive findings observed in the ANA screening test, indicating an autoimmune disease to be the cause, and aCL IgG showing 2 positive results led us to conjecture APS as the cause. The symptoms of mesenteric venous thrombosis are affected by the location and size of the thrombus. Abdominal pain, vomiting, nausea, diarrhea, and even bloody stools may be observed, and if the diagnosis and treatment are not performed early, a bowel infarction or death can occur.^[15]^ As this patient exhibited abdominal pain and no other symptom, only an abdominal CT was performed, which revealed the location of the thrombus from the superior rectal vein to the IMV confluence.

APS is an autoimmune disease in which there is a presence of specific antibodies (LA, aCL, and anti-beta2 glycoprotein-1 antibodies) that attack the phospholipids in the body. This disease occurs very rarely in children. Primary APS usually appears alone, but might also occur in combination other autoimmune diseases such as SLE. For diagnosis, both a clinical criterion (vascular thrombosis or pregnancy morbidity) and a laboratory criterion (positive LA, aCL, or anti-beta2 glycoprotein) should be satisfied. The phospholipid is a component that forms the membranes of cells constituting the endothelium. The action of the antiphospholipid antibodies attacking the phospholipids causes damage to the vascular endothelium, increasing the risk of thrombosis.^[16]^ Furthermore, several clinical studies have reported that the risk of thrombosis increases parallel with the number of antibodies in patients with APS.^[17]^ Intraabdominal manifestations include renal infarction, renal artery or venous thrombosis, esophageal or mesenteric ischemia, and spleen infarction.^[18]^

Treatment can include high-dose steroids, immunosuppressive drugs, and plasma exchange, which are used to remove aPL. However, these treatments are temporary, and once stopped, antibodies quickly recover within 3 weeks. Therefore, treatment plans aiming to remove aPL are not the first choice, except in cases of underlying diseases or life-threatening situations. If a patient diagnosed with APS develops venous thrombosis, the patient must receive anticoagulant therapy, and long-term treatment is required due to a high recurrence of thrombosis within six months after discontinuation of treatment.^[19]^ In this case, the patient started treatment with heparin at the time of hospitalization and afterward, switched to warfarin, a vitamin K antagonist, continuing treatment with INR monitoring. CT was performed every 3 months to check the resolution of the thrombus. This patient should continue to take warfarin, and long-term follow-up is required to determine the occurrence of complications.

In conclusion, according to this case report, if hepatic NRH or vascular thrombosis is seen in children, APS should be differentially diagnosed with LA and aCL, and appropriate management should be implemented.

## Author contributions

**Conceptualization:** Kyung Duk Park, Soon Chul Kim.

**Data curation:** Seung Hyung Han.

**Formal analysis:** Kyung Duk Park.

**Validation:** Seung Hyung Han, Kyung Duk Park, Soon Chul Kim.

**Writing – original draft:** Seung Hyung Han.

**Writing – review & editing:** Kyung Duk Park, Soon Chul Kim.
